# International Child Health Elective for Pediatric Residents

**DOI:** 10.1186/1824-7288-40-13

**Published:** 2014-02-05

**Authors:** Liviana Da Dalt, Giovanni Putoto, Dante Carraro, Alessandra Gatta, Eugenio Baraldi, Giorgio Perilongo

**Affiliations:** 1 Department of Woman’s and Child’s Health, Pediatric Residency Program, University of Padua, Padua, Italy; 2 Doctors with Africa CUAMM, Padua, Italy

**Keywords:** Pediatrics, Medical education, International cooperation, Global health

## Abstract

**Background:**

There are increasing evidence highlighting the importance of incorporating issues of global health into pre- and post-graduate medical curricula. Medical international cooperation is a fundamental component of strategies to include global health issues in post-graduate medical curricula.

**Methods:**

Here we describe a seven-year cooperation between the Non Governmental Organization (NGO) “Doctors for Africa CUAMM” and the Pediatric Residency Program (PRP) of the University of Padua (Italy) that offers residents a well-articulated personalized international child’s health (ICH) elective in Africa, called “Junior Project Officer”. The elective includes: a careful candidate selection process; pre-departure educational course; preceptorship in Padua and Africa, personalized learning objectives, a personalized job description, a six-month hands-on learning experience in Africa, evaluation of the experience, and formal private and open feed-backs/reports.

**Results:**

Between 2006 and 2012, 14 residents aged from 27 to 31 years, six attending the III, nine the IV and two the V year of residency completed the six-month stage in Africa. All worked in pediatric in-patient units; seven also worked in out-patient clinics, six in emergency rooms and seven in community health centers. Eleven were involved in teaching activities and four in clinical research projects. All residents claimed to have achieved their learning objectives.

**Conclusions:**

A strong partnership between the NGO and the PRP, and well-articulated personalized learning objectives and job description contributed to a successful ICH elective.

## Introduction

There is increasing pressure to incorporate issues of global health into pre- and post-graduate medical curricula in order to provide new generations of doctors with a multicultural perspective on health care [1-3]. The North American Association of Pediatric Program Directors included the commitment to improve global health education in its recent strategic plan [4] and in North America, a model curriculum in global health for pediatric residents has been proposed and implemented. Elements of domestic and/or international child health (ICH) electives are a fundamental component of this curriculum [5,6].

Despite the relevance and benefits of programs offering ICH electives, and despite numerous recommendations and guidelines on how to build them [7-12] major effort is still required to offer to residents work experience in medical international setting in countries with limited resources. The scarcity of reports describing ICH experiences could be a sign of this challenge [13-16].

Between 2006 and 2012, the Pediatric Residency Program (PRP) of the University of Padua (Italy) joined the Junior Project Officer (JPO) program promoted by the Italian non-governmental organization (NGO) “Doctors with Africa CUAMM (Collegio Universitario Aspiranti e Medici Missionari)” [17], which offers doctors-in-training the possibility of undertaking a six-month health elective in an African hospital. Here we report our seven years’ experience with this program in the hope that the articulated personalized framework of our ICH elective may be useful for other programs that offer ICH electives.

## Methods

CUAMM, the first NGO in the field of healthcare to be officially recognized in Italy, is the largest Italian body working to improve and safeguard the health of African populations. The organization has long-term goals, and provides quality healthcare services that are open to all. Its philosophy is to help Africa by creating, supporting and promoting training environments and to create within the international medical community a culture open to global health issues. It is present in seven Sub-Saharan African countries: Angola, Ethiopia, Mozambique, South Sudan, Uganda, Sierra Leone and Tanzania. It operates through volunteers, namely, health professionals such as doctors, nurses, midwives, technicians and administrative staff. These volunteers provide support to hospitals, health districts (for public health activities, mother-child care, the fight against AIDS, tuberculosis and malaria, and training), motor rehabilitation centers, nursing schools, and Medical Universities (in Uganda, Mozambique and Ethiopia).

The JPO project launched in 2002 offers doctors in training the possibility of a rotation in one of hospitals in Africa in which CUAMM volunteers work. It also aims at eliciting a vocation to work in the field of international medical cooperation. It is directed to residents in Infectious Diseases, Internal Medicine, Obstetrics and Gynecology, Pediatrics, Public Health and Surgery.

The PRP of the University of Padua is a 5-year national accredited program for post-graduate training in Pediatrics. Approximately 80% of learning activities takes place in the clinical setting, in which medicine is practiced under the supervision of a faculty member with the goal of increasing levels of responsibilities during training. The remaining learning activities are formal lectures, seminars, workshops. Residents rotate through 15 of the 25 divisions/services of the Department of Woman’s and Child’s Health of Padua and of affiliated hospitals during their first three years devoted to provide general pediatric competences; rotations last between 3 to 6 months. During the last 2 years of training, residents select elective rotations in pediatric subspecialties involving, at most, 3 divisions; each rotation lasts from 6 to 12 months.

### The elements of the JPO elective

The JPO elective is open to residents in their third year onwards. Only one resident at a time can go to Africa.

#### Selection

Candidates are selected based on their professional profile, notably pediatric knowledge, level of autonomy, manual skills and reliability, and on the results of an interview with members of the Resident Affairs Committee (RAC) and with representatives of CUAMM. The interview is designed to understand the candidate’s motivation join the project, to describe all the potentially negative and positive aspects of the experience and to try to evaluate the probability that the candidate will successfully complete the program.

#### Pre-departure educational course

After the initial screening, residents are required to attend a professional training course (“Cooperating for Health in Africa”) organized by CUAMM. This consists of six three-day modules held on a monthly base; the first is devoted to the basic principles of health cooperation with countries that have limited resources and the inspiring concepts of “Doctors with Africa CUAMM”; the second module introduces the basis of “Strengthening Health Systems” and the strategic approach adopted by the NGO in Africa. The remaining modules provide general concepts of hospital management and public health, and information about the diagnostic and therapeutic guidelines established for the main tropical infectious diseases, about the status of mother and child health in Africa, and about the more relevant critical issues and the main programs activated to address them.

#### Identification of the site in Africa and preceptorship

Once a candidate has successfully undergone the interview and the pre-departure course, the possible destination in Africa is identified, based exclusively on the presence on site of a pediatrician from CUAMM who can function as a local tutor and on safety issues related to the local social and political situation. A preceptor in Padua (one of the authors – LDD), chosen among RAC members because of experience in international medical cooperation and commitment to the JPO project, was assigned to each resident.

#### Learning objectives

Once the resident, the hospital in Africa and the CUAMM volunteer serving as local tutor are selected, the overall learning objectives of the training (see Table 1) are collegially discussed. A personalize job description is also prepared for each resident. Ideally, a simple epidemiological and/or clinical research project related to the local reality is conceived.

**Table 1:** General learning objectives of the international child health elective, and numbers of residents who declared they had achieved these goals at the end of the elective

	**Positive replies**
**Learn how to diagnose and manage the most common pediatric tropical diseases**	14/14
**Learn how to practice medicine in settings of limited resources**	14/14
**Gain insight into the world of international medical cooperation**	10/14
**Acquire empathy and experience in addressing the health care needs of underserved communities through exposure to alternative modes of heath care delivery and resource allocation**	13/14
**Develop professional values through exposure to different philosophies of medical ethics, relationships and child rights**	13/14

#### Activities in Africa

In the hospital in Africa, the resident is required to participate in all the daily activities of the service to which they have been assigned (e.g. patient’s visit, case discussion, procedures, meetings with nurses and families). All his/her activity is carried out under the supervision of the local tutor in Africa.

#### Evaluation

The 6-month ICH elective is counted as any other rotation that is mandatory for residents during their training. Consequently, it is subject to the usual PRP evaluation process, which foresees evaluation of the residents by tutors, and of the quality of the rotation and of the tutors’ performance by residents, based on validate questionnaires [18]. Therefore, the RAC of the PRP in Padua evaluates residents, tutors, the quality of rotations, and the six months spent in Africa. In addition, upon their return to Padua, residents are requested to complete another questionnaire specifically designed to evaluate their experience in Africa, which is then discussed during an oral interview with RAC members and a representative of the NGO. Finally, residents are invited to present their experience during an ad hoc seminar, ideally attended by all other residents and the faculty of the Hospital in Padua.

#### Practicalities

CUAMM provides participants with the necessary insurance coverage for personal and professional risks, and assistance in applying for VISA and work permits, as required, and lodging on site in residences rented by CUAMM. The residency program covers travelling costs, whereas residents must pay for all other expenses they incur in Africa from the salary they continue to receive from the University.

### Other components

#### Memorandum of understanding (MoU)

The JPO program is governed by a memorandum of understanding, signed by CUAMM and the University of Padua, according to which the NGO and the University undertake to cooperate to improve the health status of the population in Africa by making available their own specific resources and competences.

#### Site visit

Before the JPO project started in October 2005, RAC members visited the hospitals chosen for the project. The aims of the site visit was to meet the local political and health authorities, to describe the PRP, to see the hospitals and their facilities in order to acquire an overall sense of the environment where residents are expected to work.

## Results

**Figure 1:**
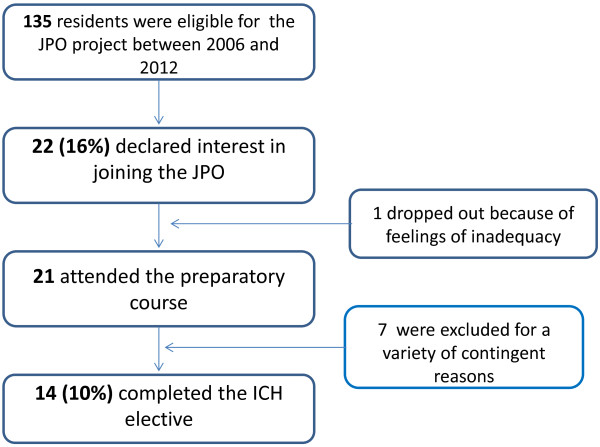
**Junior Project Officer (JPO).** The selection process of residents used by the Pediatric Residency Program of Padua University. Selection of residents for the Junior Project Officer (JPO) International Child Health Elective run by the Pediatric Residency Program of Padua University. ICH = International child health.

Between 2006 and 2012, of the 135 residents who participated in the PRP and were eligible for the JPO, 22 declared their interest in joining the project and attended the preparatory course. One dropped out because she did not feel psychologically prepared, and seven were unable to go to Africa for contingent reasons. Thus, 14 residents went to Africa (Figure 1). All successfully completed a six-month stage; four in St. Luke’s Hospital (Wolisso, Ethiopia), and 10 in Central Hospital (Beira, Mozambique). The features of the two hospitals are listed in Table 2. No temporal gaps elapsed between one stage and another and stages did not overlap.

**Table 2:** Main features of the Wolisso (Ethiopia) and Beira (Mozambique) hospitals

	**Beira (Mozambique)**	**Wolisso (Ethiopia)**
**Hospital authority**	State	Catholic Church (CUAMM)
**Total number of beds**	730	200
**Total number of pediatric beds**	128	55
**Pediatric units/services**	General Pediatrics	General Pediatric
	Infectious Diseases	Emergency room
	Malnutrition	
	Neonatology	
	Emergency room	
**Medical and surgical units**	Internal Medicine	Internal Medicine
	Surgery	Surgery
	Obstetrics & Gynecology	Obstetrics & Gynecology
	Orthopedics	Intensive Care
	Intensive Care	Rehabilitation
**Pediatric hospital admissions (year)**	9,000	2,500
**Pediatric ER care visits**	30,000	10,000
**Newborns per year**	6,000	3,000
**University affiliation**	With the Catholic University of Beira	No

All 14 residents but one were women (essentially reflecting the female prevalence in our program - 85% of the residents). Their age ranged from 27 to 31 years; six were attending the III, nine the IV and two the V year of residency. While in Africa, all worked in pediatric in-patient units; seven were also involved in out-patient clinics, six in emergency rooms and seven in territorial health centers. Eleven were also involved in teaching activities for local nurses and doctors in-training, and four carried out a clinical research project while in Africa.

As reported in Table 1, all residents declared they had achieved the learning objectives of the ICH elective, except four who felt they had not gained “deep insight into the world of international cooperation” because of the short duration of their permanence abroad. It could be also hypothesized, that these four residents were overwhelmed by the pressure of treating patients, and they failed to see the overall picture of the environment in which they were working. In general, residents appreciated all components of the program; in fact all would repeat the experience and would recommend it to others (Table 3). The two negative replies regarding the adequacy of the tutorship in Africa were due, in one case, to personality issues between the resident and tutor, and in the other case, the tutor had to leave Africa and no suitable substitute was found. Five residents declared they encountered problems related to the different life style; one found the situation too difficult and stressful due to the many deaths she assisted mainly related to the lack of resources commonly available in the place she was used to work); two had communication problems due to languages barriers; and one experienced personal security issues after suffering a street theft. Upon their return to Padua, all residents delivered a seminar describing their experience. One participant joined CUAMM after completing her residency, and another had a second experience of international medical cooperation in an African hospital.

**Table 3:** Questionnaire used by the residents to evaluate their experience in Africa and main results

**Questions**	**% (Positive replies)**
**Was the preparatory course helpful?**	14/14
**Were the learning objectives of your stay in Africa clear?**	13/14
**Was your personalized job description sufficiently clear and respected?**	13/14
**Was the setting where you went to work well described before your departure?**	12/14
**Do you think that the ICH elective:**	
**Prepared you sufficiently?**	13/14
**Adequate time-wise?**	12/14
**Enabled you to grow professionally?**	13/14
**Was the perceptorship that was provided to you in Padua important?**	14/14
**Was the tutorship you had in Africa adequate?**	12/14
**Do you think you have achieved the learning objectives?**	See Table 1
**Do you think it was an added value to have been part of a CUAMM project?**	14/14
**Are you happy with this experience?**	14/14
**Would you repeat it?**	14/14
**Would you recommend it to others?**	14/14
**Did you suffer from:**	
**The different life style in Africa?**	05/14
**Too difficult and stressful situations?**	01/14
**Communication problems?**	02/14
**Personal security issues?**	01/14
**Any diseases?**	--/14

## Discussion

To our knowledge, this is the first article to describe the strategic framework of an ICH elective for students enrolled in a European PRP. Furthermore, no other Italian pediatric residence programs offer similar experience based on such an articulated framework. During the seven years that the PRP of Padua University participated in the JPO project, there was always a candidate for the ICH elective. Moreover, all the 14 residents who participated in the elective completed the six-month stage abroad, without having encountered any major problems, which is remarkable given the duration of the stage. Notably, all felt they had undergone an important human and professional experience. Therefore, the JPO project, as carried out by the PRP of Padua University, is a useful example of how to offer ICH electives to pediatricians in training (Table 4).

**Table 4:** Key components of the junior project offer elective

**Pre-departure**	**The partnership with CUAMM, a non-governmental organization operating in countries with limited resources, that focuses on the role of education**
	**The MoU signed by the University of Padua (to which the PRP belongs) and CUAMM**
	**The site visit to the Africa hospitals**
	**The selection process of the candidates**
	**The pre-departure course**
	**The definition of the learning objectives and the personalized job description**
**During the elective**	**The preceptorship both in Padua and in Africa**
	**The active involvement in all clinical activities**
	**The evaluation process**
**At the end of the elective**	**The feed-backs**
	**The summary report presented to the PRP, the residents and the hospital staff**

Legend: ICH: International Child Elective; MoU: Memorandum of understanding; PRP: Pediatric Residency Program.

A cornerstone of our program was careful selection of residents for the ICH elective. Although aware that the residents would work in a structured medical environment and under supervision, RAC members were concerned that residents might not be able to continue if their motivations, their psychological profile, their level of professional and human maturation and their expectations were not in line with the reality they would face in Africa. To obviate this, all JPO fellows attended a preparatory course, which, upon their return, they recognized to be very important. Similarly, they also recognized the importance of having established precise educational objectives and general work descriptions before their departure (see Table 3). They declared that that these two elements helped to keep them focused on the ultimate aims of their ICH elective and on their motivation to take part in the program.

Approval of an ICH elective depended on the availability of motivated tutors both in Padua and in Africa. The task of the tutor in Padua, who had undergone a lengthy ICH elective in Africa and thus served as a role model, was to understand the residents’ vocation, to guide them through the thinking process underlying their decision to participate in the ICH and finally to provide them with human and professional “long distant” support (via internet) while in Africa. As witnessed by the residents’ feedbacks, this tutorship was greatly appreciated. The tutorship in Africa was considered equally effective. In the two cases in which the resident did not experience a positive tutorship in Africa, the situations were carefully monitored, and the problems did not prevent the residents from completing their ICH elective and reporting positive comments.

The MOU of the University of Padua, within which the PRP operates, is a key factor of our IHC elective. In fact, in this MOU the University of Padua endorses the CUAMM-PRP partnership and thus recognizes the educational value of the JPO project and its impact on society as a whole. Thus, the CUAMM-PRP partnership has the status of an academic endeavor and consequently sets rigorous standards for its development and implementation. The MOU served also to provide the administrative and legal elements necessary to run the project in Padua. Our experience suggests that PRPs offering ICH electives should have a formal, officially recognized (by the authorities governing the PRP) relationship with international medical organizations that are well positioned in countries with limited resources. Such organizations are able to facilitate the access of residents to hospitals in Africa, identify local expert tutors and provide all the resources necessary to support the residents’ presence in Africa. Ideally, they should share CUAMM’s commitment to education.

We are aware that such an ICH does not fulfill all the requirements of a complete pediatric global health education [5]. Undoubtedly, much more should be done in terms of theoretical education and practice and, at least in Italy, in terms of official recognition of an educational curriculum in global health. In fact, in Italy the initiative described herein was possible only because of the University of Padua’s independent decision to endorse it. On the other hand we believe that it is quite a unique feature of this project the fact that the residents can spend a faire amount of time at the overseas site and thus getting a much better sense of global health that most of other programs can do which constrict the residents to 2- to 4-week overseas visits [13-15].

Finally, if one of the reasons to offer an ICH was to solicit professional vocations in the field of international medical cooperation, it is noteworthy that two of the residents who participated in the ICH are currently involved in programs of international medical cooperation (and one of them with CUAMM).

In conclusion, we decided to make the pediatric community aware of the model we used to offer an ICH elective with the aim of stimulating a debate on this matter and of generating criticisms and ideas. This, in turn, might encourage other PRPs to offer ICHs to residents. Indeed, more should be done to implement the culture of medical global health in medical post-graduate programs. It is important to raise future generations of doctors with the concept that we live in a world of strong inter-dependency where everyone should practice medicine in the part of the world in which they work but with their gaze directed to wider horizons.

## Abbreviations

ICH: International child health; JPO: Junior project officer; MoU: Memorandum of understanding; NGO: Non Governmental Organization; PRP: Pediatric residency program; RAC: Resident affairs committee.

## Competing interests

All authors declared no competing interest with the contents of the manuscript.

## Authors’ contributions

LDD: Among the ones conceiving the MPO project for the Pediatric Residency Program of Padua; intervened in collecting the data of the experience, analyzing them and in writing the paper; preceptor in Padua for the residents in Africa. GPer & EB: Past and present chairman of the Pediatric Residency Program of Padua, they intervened in supporting the JPO project, in analyzing the data and in writing the manuscript. DC & GPut: the Founder of the JPO project in Italy; they intervened in supporting the JPO from the CUAMM size, in supervising the analysis of the data and of the manuscript. AG: Chair of the organizing committee of the JPO project, she intervened in developing the capturing forms for analyzing the data regarding the experience, in data managing and in formulating the first set of analysis. All authors read and approved the final manuscript.

## Authors’ information

DC, MD, Director, Doctors with Africa CUAMM, Padua, Italy. EB, Full Professor of Pediatrics, University of Padua, senior member of the Resident Affair Committee; Chair of the Pediatric Residency Program of Padua University since October 2012. LDD, Associated Professor of Pediatrics, University of Padua, Chair of the Pediatric Divisions, expert in post-graduate medical training; Vice-Chair and senior member of the Resident Affair Committee of the Paediatric Program of Padua. AG, JPO Project Manager – Human Resources Department, Doctors with Africa CUAMM, Padua, Italy. GPer, Full Professor of Pediatrics, University of Padua, Director of the Department of Woman’s and Child’s Health of the University of Padua, Italy; Chair of the Pediatric Training Program from 2005 to September 2012; presently member of the Resident Affair Committee. GPut, MD, Head of Planning, Doctors with Africa CUAMM, Padua, Italy.
